# Increasing the efficiency of study selection for systematic reviews using prioritization tools and a single-screening approach

**DOI:** 10.1186/s13643-023-02334-x

**Published:** 2023-09-14

**Authors:** Siw Waffenschmidt, Wiebke Sieben, Thomas Jakubeit, Marco Knelangen, Inga Overesch, Stefanie Bühn, Dawid Pieper, Nicole Skoetz, Elke Hausner

**Affiliations:** 1 Institute for Quality and Efficiency in Health Care, Cologne, Germany; 2 Department 2 (Infectious Disease Epidemiology), Public Health Agency of Lower Saxony, Hanover, Germany; 3 Institute for Research in Operative Medicine, Herdecke University, Witten, Germany; 4 Faculty of Health Sciences Brandenburg, Brandenburg Medical School, Institute for Health Services and Health System Research, Rüdersdorf, Germany; 5 Brandenburg Medical School, Center for Health Services Research Brandenburg, Rüdersdorf, Germany; 6 Evidence-Based Medicine, Department I of Internal Medicine, Faculty of Medicine, University Hospital Cologne, University of Cologne, Cologne, Germany

**Keywords:** Systematic reviews, Study selection, Methodology

## Abstract

**Background:**

Systematic literature screening is a key component in systematic reviews. However, this approach is resource intensive as generally two persons independently of each other (double screening) screen a vast number of search results. To develop approaches for increasing efficiency, we tested the use of text mining to prioritize search results as well as the involvement of only one person (single screening) in the study selection process.

**Method:**

Our study is based on health technology assessments (HTAs) of drug and non-drug interventions. Using a sample size calculation, we consecutively included 11 searches resulting in 33 study selection processes. Of the three screeners for each search, two used screening tools with prioritization (Rayyan, EPPI Reviewer) and one a tool without prioritization. For each prioritization tool, we investigated the proportion of citations classified as relevant at three cut-offs or STOP criteria (after screening 25%, 50% and 75% of the citation set). For each STOP criterion, we measured sensitivity (number of correctly identified relevant studies divided by the total number of relevant studies in the study pool). In addition, we determined the number of relevant studies identified per single screening round and investigated whether missed studies were relevant to the HTA conclusion.

**Results:**

Overall, EPPI Reviewer performed better than Rayyan and identified the vast majority (88%, Rayyan 66%) of relevant citations after screening half of the citation set. As long as additional information sources were screened, it was sufficient to apply a single-screening approach to identify all studies relevant to the HTA conclusion. Although many relevant publications (*n* = 63) and studies (*n* = 29) were incorrectly excluded, ultimately only 5 studies could not be identified at all in 2 of the 11 searches (1x 1 study, 1x 4 studies). However, their omission did not change the overall conclusion in any HTA.

**Conclusions:**

EPPI Reviewer helped to identify relevant citations earlier in the screening process than Rayyan. Single screening would have been sufficient to identify all studies relevant to the HTA conclusion. However, this requires screening of further information sources. It also needs to be considered that the credibility of an HTA may be questioned if studies are missing, even if they are not relevant to the HTA conclusion.

## Background

Systematic literature screening is a key component in systematic reviews and health technology assessments (HTAs). Stringent requirements exist for the transparency of the study selection process and the reliability of results, aiming to avoid the omission of relevant evidence with a subsequent risk of bias endangering the validity of conclusions [1–3].

### Prioritization using text mining

A systematic literature search generally yields thousands of hits, making manual screening resource intensive or even unfeasible. Several Internet-based screening tools such as Abstrackr [4], Rayyan [5], Covidence [6], and EPPI Reviewer [7] have been developed over the past years to make screening more efficient and are widely used. To select screening tools for our study, we evaluated the advantages and disadvantages of tools that prioritize references using text mining in a prestudy in 2016 [8]. We then selected Rayyan and EPPI Reviewer; as in our opinion, these tools are suitable for use in daily practice and appear to be sustainable. Both apply a machine-learning algorithm to prioritize the order in which citations are presented for screening. Details on the different screening tools available at that time and our assessment can be found in the protocol [9]. The ranking of citations continuously improves as screening progresses, with more and more manual decisions being available from which the algorithm can learn. Since the start of our study, other prioritization tools have been developed (e.g., DistillerSR [10]), but could not be considered.

### Single-screening approach

When screening bibliographic search results, study selection is generally performed as a two-step process conducted by two persons independently of one another (double-screening approach) [11, 12]. However, this approach is resource intensiv, which can pose a problem, as systematic reviews and HTAs generally need to be completed within a defined period with a limited budget [1, 2]. A single-screening approach might seem meaningful to reduce the workload as instead of two screeners, only one screener would have to scrutinize all title/abstracts and full texts. However, the few studies investigating this approach (published up to 10/2018 and included in a systematic review [11]) did not provide sufficiently robust evidence to recommend single instead of double screening as the standard approach for study selection. Furthermore, a study from 2020 showed that single screening carries a high risk of missing a large proportion of relevant studies [13].

## Objectives

The aim of the present analysis was to examine the following questions related to the process of study selection from the results of the bibliographic search:

- ▪ Question 1: Can the use of the Rayyan or EPPI Reviewer tools for prioritizing the results of study selection increase efficiency?
- ▪ Question 2: How accurately does a single-screening approach identify relevant studies?

According to the protocol, a third question was to be investigated [9], but this question was omitted. Please see the section “protocol deviations” for the corresponding reasons.

## Methods

We conducted a prospective analysis of study selection processes based on HTAs of drug and non-drug interventions performed by the German Institute for Quality and Efficiency in Health Care (IQWiG). The study protocol was published a priori [9].

For the bibliographic search, study selection using a single-screening approach was tested by means of the original searches in the HTAs. Each search was eligible for inclusion in the analysis. There was no restriction with regard to the study type considered. If an HTA involved more than one search (e.g., in HTAs on screening tests, one search for studies on diagnostic accuracy and a second search for studies on the screening algorithm), each study selection process was to be analysed separately.

**Fig. 1:**
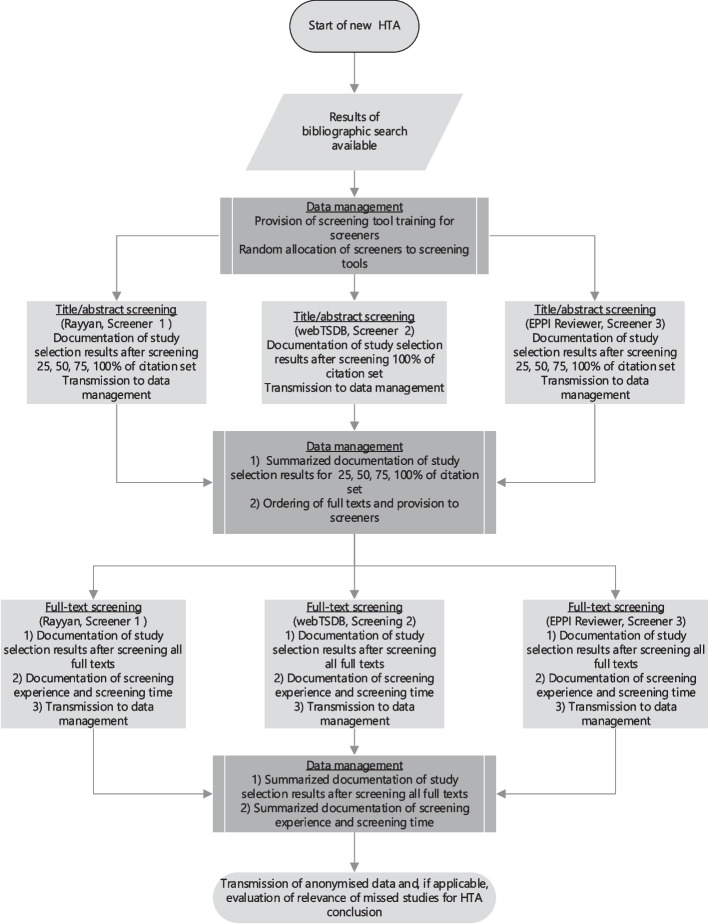
Workflow and data management process

Figure 1 illustrates the workflow within the study.

All HTAs and the corresponding searches involved three screeners using two screening tools with prioritization (Rayyan, EPPI Reviewer) and one without prioritization (IQWiG’s internal screening tool “web Trial Selection Data Base,” web TSDB). Two of the three screeners—usually the project leader and another researcher—were part of the IQWiG project group that conducted the HTA. The third screener was either an IQWiG researcher not involved in the HTA or a researcher from the the Evidence-based Medicine Unit at the University of Cologne commissioned by IQWiG for this specific task. All screeners received practical training by IQWiG information specialists on the functions of the three screening tools as well as training materials. They also were supported by the project group if they had any questions regarding the tools.

One question of interest is the probability that the study pool of a single screener includes all relevant studies. This probability is estimated by the frequency of screenings in which all relevant studies were found, relative to the total number of screenings. A one-sided Wilson 95% confidence interval (CI) was calculated to estimate this probability. If the lower limit of the CI was greater than 90%, a single screener was considered sufficient for screening because the probability of finding all relevant studies was estimated to be greater than 90%.

The planned number of at least 33 screenings (11 searches screened by three screeners each) allows the lower limit of the CI to reach a value of 92.4% if all 33 screenings identify the relevant studies (i.e., if no screener makes an error). If one error occurred among the 33 screenings, the 90% limit would not be reached because the lower limit of the CI would be 87.5%.

On the basis of this sample size calculation, we consecutively included 11 searches and study selection processes from the start of our project. Before the selection process started, each screener was given the protocol with the study inclusion and exclusion criteria, as well as potentially relevant study publications and systematic reviews and HTAs, if available.

The three screeners per study selection process then independently screened all citations on the title and abstract level using the three screening tools. All full texts classified as potentially relevant by at least one screener were ordered, and each screener received only those texts he or she had requested. No consensus procedure was performed at the title/abstract or full-text level for studies with inconsistent classifications. The results of the selection process were recorded and evaluated separately for each screener.

On completion of the selection process, studies classified as relevant by at least one screener were allocated to the study pool and forwarded to the project group for further assessment. Further information sources (e.g., searches in study registries, scanning of reference lists) used in addition to bibliographic databases were screened in the conventional way (i.e., one person performed the screening and a second person checked the results), and if applicable, additional relevant studies were added to the study pool. The different project groups then further processed the study pool (e.g., extraction of study characteristics, assessment of risk of bias). In this project phase, it was decided whether certain studies should be excluded from further assessment (e.g., due to a lack of relevant outcome data); if this was the case, they were removed from the study pool retrospectively. The reference standard comprised only those publications and studies identified in the bibliographic search and classified by all three screeners and the project group as relevant. As stated, the final study pool could also include additional relevant studies identified by searches in further information sources. These studies were not included in the reference standard, but were included in the evaluation of the relevance of missed studies to the HTA conclusion.

Data collection and analysis were anonymized and blinded, i.e., it was not disclosed which screener yielded which study pool with which tool.

### Outcomes

The following outcomes were analyzed retrospectively to investigate the question as to whether the use of prioritization tools increased efficiency (Question 1):

- Proportion of citations classified as relevant at three cut-off or STOP criteria (after screening 25%, 50%, and 75% of the citation set)
- “STOP criteria” indicates one of the three pre-specified cutoffs (25, 50, or 75%) at which the selection process could be stopped.

The following outcomes were analyzed to investigate the question as to how accurately each single screener identified relevant studies (Question 2):

- Number of relevant publications and studies identified. “Relevant” means either that all studies of the reference standard were identified or that the studies not identified were irrelevant to the HTA conclusion. “Relevant to the HTA conclusion” means that the overall conclusion about the benefit of a treatment option would not change.
- Sensitivity (number of correctly identified relevant studies divided by the total number of relevant studies in the study pool).
- Number of missed studies classified as “not relevant”: To evaluate relevance, changes in the study pool and a subsequent potential change to the HTA conclusion were assessed for each outcome. Firstly, for all outcomes to which the missed study contributed data, we checked whether the estimated effects would change from significant to non-significant or vice versa if the study was omitted in the analysis. Secondly, we evaluated whether any changes in effects would change the HTA conclusion. If no such change was found for any outcome, the studies missed in the selection process were classified as “not relevant.”

### Information synthesis and analysis

All data were analyzed and presented using descriptive statistics. As each search involved three screeners and individual screeners could screen more than once, data dependencies existed. The sample size calculation roughly followed the confidence intervals (CIs) that could be reached under the assumption of data independency (which, as stated, was not fulfilled) for the analyses described below.

For Question 2, the probability that the study pool of a single screener included all relevant studies was estimated by means of the relative frequency of selection processes yielding all relevant studies in relation to all selection processes. A one-sided CI (Wilson method) was calculated for this proportion. If its lower limit was more than 90%, it was assumed that the single screener would yield a study pool of all relevant studies with sufficient certainty. The planned number of 33 screenings allowed a lower limit of 92.4% to be reached if all 33 contained all relevant studies (i.e., if no screener made a mistake). If one mistake was made in 33 processes, then the 90% CI was missed, as the lower limit was 87.5%.

Previous screening experience was considered as a potential effect modifier. By means of a self-assessment questionnaire, the screeners were asked a priori to classify their previous screening experience into 1 of 3 categories: “little experience” (< 3 screenings), “average experience” (3 to 10 screenings), and “great experience” (> 10 screenings). If further potential effect modifiers were identified during the analysis, they were also considered. However, no such modifiers were identified.

Due to data protection reasons, data collection and analysis were anonymized and blinded, meaning that it was not known which screener yielded which study pool with which tool.

### Protocol deviations

According to the protocol, a third question was to be investigated: “Which advantages or disadvantages (e.g., shortened screening time or increase in the number of full texts ordered) does a single-screening versus a double screening approach have?” [9]. Due to limited resources, this additional comparison was not possible. Therefore, data on the outcomes “number of full texts ordered” and “time required for study selection” were collected for the 33 single screenings, but could not be compared with the double-screening approach and are not presented here.

Moreover, several analyses could not be performed as originally planned as, due to the study design (anonymized and blinded data collection and analysis), it was only possible to report proportions, not numbers. This affected the following outcome specified in the protocol: the number of publications not needed to be screened with a STOP criterion. In addition, we did not calculate specificity (number of correctly identified irrelevant studies divided by the total number of irrelevant studies) as we only considered the number of relevant studies not identified to be important. Finally, we performed a post hoc evaluation: before we assessed the potential change to the HTA conclusion, we checked whether the missing studies would have been identified through further information sources used in the HTAs.

## Results

### Search results

Eleven bibliographic searches and study selection processes from 10 HTAs were conducted between June 2018 and March 2020 (Table 1 and Appendix 1). The citation set included 9196 citations [min 77, max 1571 per HTA], a mean of 12% [min 1%, max 28%] were ordered in full text and 4% [min 0.2%, max 17%] were included after full-text screening.

**Table 1:** List of HTAs and results of bibliographic searches included in the analysis

No	HTA No./study type	Title	Search date^a^	Number of hits^a^	Number of relevant studies (publications^b^)
1	N18-01 [14] RCT	Synchronous balneo-phototherapy for atopic eczema	01.06.2018	337	1 (1)
2	N18-02 [15] RCT	Tumour-treating fields in addition to current standard therapy for glioblastoma as first-line treatment	04.01.2019	77	1 (7)
3	S18-01 [16] Non-RCT	Newborn screening for sickle cell anaemia	28.08.2018	1460	1 (1)
4	HT18-04 [17] RCT	Seasonal affective disorder: Do non-drug interventions such as light and vitamin therapy lead to better results?	10.01.2019	553	20 (23)
5	N18-03 [18] RCT	Mandibular advancement device in mild to moderate obstructive sleep apnoea in adults	27.11.2018	548	34 (52)
6	A18-83 [19] RCT	Ezetimibe for the prevention of cardiovascular events	28.01.2019	1571	6 (26)
7	S18-02 [20] Search 1 Non-RCT	Newborn screening for 5q-linked spinal muscular atrophy	22.02.2019	677	2 (2)
8	S18-02 [20] Search 2 Non-RCT		14.03.2019	501	1 (1)
9	N19-01 [21] RCT	Data-supported timely management in cooperation with a physician-staffed centre for telemedicine in advanced cardiac failure	29.04.2019	1567	4 (12)
10	N19-02 [22] RCT	Autologous chondrocyte implantation in the knee joint	19.09.2019	695	14 (25)
11	HT19-02 [23] RCT	Pain in endometriosis: Do other procedures instead of painkillers also help?	24.03.2020	1210	9 (10)
			Total	9196	93 (160)

^a^Number of hits and search date of the initial search. Search updates were conducted, but not considered in the analysis

^b^Number of publications and studies identified by the bibliographic search (without search updates)

*Question 1: Can the use of the Rayyan or EPPI Reviewer tools for prioritizing the results of study selection increase efficiency?*

Of the 22 single screenings planned per prioritization tool, due to technical problems, 10 were conducted with EPPI Reviewer and 7 were conducted with Rayyan (Table 2). The missing 5 screenings could not be included, mainly because the reviewers did not send their results after screening 25, 50, and 75% of the citation set. Overall, EPPI Reviewer identified relevant citations earlier in the screening process, resulting in 88% sensitivity for relevant citations after screening half of the citation set (Table 2 and Appendix 2). The corresponding sensitivity for Rayyan was only 66%. In 5 out of 10 screenings, EPPI Reviewer identified all relevant citations after screening half of the citation set; for Rayyan this was only the case in 1 out of 7 screenings. Although EPPI Reviewer appeared to be clearly superior here, it should be noted that in two screenings, this tool identified only 43% and 60% of the relevant citations after screening half of the citation set.

**Table 2:** Results of potential STOPs for prioritization

Tool	Number of screenings^a^	Proportion of relevant citations: STOP after 25% mean [min–max]	Proportion of relevant citations: STOP after 50% mean [min–max]	Proportion of relevant citations: STOP after 75% mean [min–max]
EPPI	*n* = 10	76% [14–100%]	88% [43–100%]	93% [71–100%]
Rayyan	*n* = 7	53% [0–83%]	66% [0–100%]	75% [0–100%]

^a^Due to technical problems (e.g., prioritization was not triggered or export of citations was omitted); in 5 screenings, prioritization was not applied as planned, affecting 1 and 4 study selection processes with EPPI Reviewer and Rayyan, respectively

As only two non-RCT searches were included in Rayyan and three in EPPI Reviewer, it was not possible to analyse the potential effect modifier “study type, i.e., no clear pattern could be identified for better prioritization of RCTs or non-RCTs for either tool.

*Question 2: How accurately does a single-screening approach identify relevant studies?*

For 6 out of 11 study selection processes, 63 publications (29 studies) were not identified by the single screenings (Table 3). Overall, the median proportion of missed studies and publications was 0%, but the range was wide [min 0–max 100%] (Table 4).

**Table 3:** Overview of missed publications/studies and their relevance to the HTA conclusion

	**Number of publications not identified**	**Number of studies not identified**	**Number of studies identified via other sources**	**Number of studies not identified by any other source**	**Relevance of these studies to HTA conclusion**
A18-83	12	4	4	0	-
S18-01	1	1	1	0	-
S18-02 Search 1	0	0	0	0	-
S18-02 Search 2	0	0	0	0	-
N18-01	0	0	0	0	-
N18-02	5	0	0	0	-
N18-03	20^a^	10^a^	6	4	none
N19-02	11	4	4	0	-
N19-01	6	2	2	0	-
HT18-04	8	8	7	1	none
HT19-02	0	0	0	0	-
**Total**	**63**^**b**^	**29**^**c**^	**24**	**5**^**d**^	**-**

^a^Two publications/studies were only formally included and not evaluated and are therefore not counted here

^b^Title/abstract level: 39; full-text level: 24

^c^Title/abstract level: 16; full-text level: 13

^d^Title/abstract level: 4; full-text level: 2 (one study was excluded at both the title/abstract and the full text) level)

**Table 4:** Median proportion of missed studies and publications

		Median proportion missed		Sets of screenings	Min. in %		Max. in %	
		Ti/Ab	Full text		Ti/Ab	Full text	Ti/Ab	Full text
**Total**	**Studies**	**0%**	**0%**	**33**	**0%**	**0%**	**100%**	**100%**
	**Publications**	**0%**	**0%**	**33**	**0%**	**4%**	**100%**	**100%**
Little experience (< 3 previous screenings)	**Studies**	0%	0%	3	0%	0%	0%	8.3%
	**Publications**	0%	0%	3	0%	0%	8.3%	33.3%
Average experience (3–10 previous screenings)	**Studies**	0%	0%	14	0%	0%	100%	100%
	**Publications**	0%	2%	14	0%	0%	100%	100%
Great experience (> 10 previous screenings)	**Studies**	0%	0%	16	0%	0%	17%	21.7%
	**Publications**	2.15%	6.3%	16	0%	0%	28.8%	32.7%

*Ab* abstract, *Ti* title

We retrospectively checked whether these studies would have been identified via the other information sources used in the HTAs (i.e., via scanning of reference lists or searches in study registries). This was the case in 4 of the 6 study selection processes. For the other two projects, the statistician performed new meta-analyses and another researcher assessed the relevance of the missing studies to the HTA conclusion. In both cases, the conclusion would not have changed if the missing studies had been included.

Sixteen screeners were very experienced, 14 had average experience and 3 had little experience (Table 5). Due to the low number of screeners in the last group, no comparison with more experienced screeners was possible. The number of screeners in the groups with average and great experience was similar (14 vs. 16). Surprisingly, after adjusting for the difference in the number of screeners, the group with great experience falsely excluded 2.2 times more studies. The reasons were not further investigated.

**Table 5:** Incorrect exclusion of publications and studies according to screener experience

Experience of the 33 screeners	Number of publications not identified	Number of studies not identified
Little (< 3 previous screenings): *n* = 3	4	1
Average (3–10 previous screenings): *n* = 14	20^a^	8^a^
Great (> 10 previous screenings): *n* = 16	39^a^	20^a^
Total	63	29

^a^Two publications/studies were only formally included and not evaluated and are therefore not counted here

## Discussion

*Question 1: Can the use of the Rayyan or EPPI Reviewer tools for prioritizing the results of study selection increase efficiency?*

Overall, EPPI Reviewer performed better than Rayyan and identified the vast majority (88%) of relevant citations after screening half of the citation set. However, this finding seems insufficient to decide that only half of the citation set need to be screened if this prioritization tool is used. After screening three quarters of the citation set, the proportion of relevant citations identified in EPPI Reviewer (93%) is probably sufficient to recommend stopping screening, but at this late stage, the amount of resources that can be saved is limited and it is questionable whether accepting a remaining number of missed studies is justified. With regard to our results, it should be noted that a more positive evaluation would presumably have been possible if we had applied the same approach to Question 1 as to Question 2 (check whether missed studies were identified in other sources and check whether the remaining missed studies were relevant to the HTA conclusion).

### Comparison with previous research

Other validation studies on prioritization [24–27] achieved slightly better results. However, some of the methods applied were only of limited comparability to our study. For instance, we considered fixed cutoffs after screening 25%, 50%, or 75% of the citation set. Thomas 2021 built the Cochrane RCT Classifier predicting a score, with a higher value representing an increased likelihood that a given citation reports an RCT. Screening with the EPPI Reviewer stopped when a certain score was reached [26]. The Norwegian Institute of Public Health (NIPH) switched to a single-screening approach after the number of relevant citations identified decreased (“until the inclusion rate flattens” [25]). The method used in Tsou 2020 was comparable to our approach [27]. They compared Abstrackr and EPPI Reviewer in 10% increments to analyze screening prioritization in systematic reviews and concluded that the two tools “performed well, but prioritization accuracy varied greatly across reports “ and that “prioritization functionality is a promising modality offering efficiency gains “ [27].

### Limitations

Firstly, compared with similar methodological studies, the number of hits and relevant studies yielded by the searches was relatively low (a mean of 763 hits [77–1567] with a mean of 8 relevant studies per search [1–34]). Comparable studies were conducted with much larger citation sets, providing more relevant and irrelevant citations for the learning process in the prioritization system. For example, Tsou 2020 analyzed screenings with 226 to 9038 hits (4–104 relevant studies); prioritization often, but not always, worked better with larger citation sets [27]. NIPH analyzed screenings with 14,000 hits (number of relevant studies not reported) [25].

Secondly, due to the study design, it was not possible to analyse whether the number of hits to be screened or the size of the final study pool had an impact on prioritization. Thirdly, the comparison of the prioritization tools was hampered by the fact that only a relatively small number of screenings were possible with Rayyan (*n* = 7) due to technical problems. In addition, we were not able to investigate further in which cases the prioritization tool gave better or worse performance results (e.g., drugs vs. non-drugs). Therefore, we do not know whether the performance might be topic-related or whether there are other reasons. Finally, we did not take into account whether the differences in the design of the study selection tools had an impact on the number of correctly identified relevant studies.

*Question 2: How accurately does a single-screening approach identify relevant studies?*

In our analysis, as long as additional information sources were screened, it was sufficient to apply a single-screening approach for the bibliographic search results to identify all studies relevant to the HTA conclusion. Ultimately, even though many relevant publications (*n* = 63) and studies (*n* = 29) were incorrectly excluded, this approach did not change the overall conclusion in any HTA. Despite this finding, this does not necessarily mean that it would always be appropriate to perform single screening. For example, the obvious absence of studies in an HTA may raise doubts about its credibility and suitability for decision-making in health care. To subsequently determine whether missed studies would have changed the overall conclusion might not be a feasible approach, as this may require resources that may be greater than those required for double-screening. It should also be noted that in the present analysis, only studies that were not found via other sources (e.g., screening of reference lists, searches in study registries) were analyzed for relevance to the HTA conclusion. Twenty-four of 29 studies that were missed by single screening were found via these sources; meaning that, in addition to bibliographic databases, searches in other sources would become a highly important component of information retrieval. Screening these sources might therefore require more resources.

To classify the proportion of resources that could potentially be saved, it is important to know that IQWiG has already been able to substantially reduce screening resources in bibliographic searches by applying more precise search strategies and filters, thus avoiding an excessive number of hits. In their analysis of resource use during systematic review production, Nussbaumer-Streit 2021 noted that while study selection seemed to be very resource intensive, project management and coordination actually needed the largest proportion of production time [28], meaning that reducing resources for study selection would have only a limited impact on the overall reduction of resources. Moreover, further information sources have become more and more important such as study registries [29] and full clinical study reports (in Germany, these reports must be provided by drug manufacturers for HTAs of new drugs [30]).

### Comparison with previous research

The present study showed comparable results to our previous systematic review investigating single versus double screening (Waffenschmidt 2019 [11]), where the median proportion of missed studies was 5% (range 0 to 58%). Only two other studies have assessed the relevance of missed studies for conclusions: Shemilt 2016 [31] also investigated single screening with prioritization tools and found no change in conclusions. Pham 2016 [32] analyzed the impact of 4 methodological shortcuts (including single screening) on systematic reviews and found that single screening resulted in substantial changes in conclusions in 3 out of 6 screenings.

### Limitations

Firstly, instead of comparing a conventional double-screening approach with a single-screening approach, we summarized the screening results of the three single screeners as a reference standard. The conduct of such a comparison would have required substantial resources (e.g., larger sample size, more screeners) and was therefore not feasible. Secondly, screeners can become aware of missing studies through additional channels while working on a project (we did not check whether this was the case). In reality, the proportion of unidentified studies/publications might therefore be even smaller. It is also possible that the discussions within the project group about screening affected study selection, meaning that some screeners may have screened better than they normally would have. Thirdly, with regard to screener experience, due to the low number of screeners with little experience and the inconclusive results for screeners with average and great experience, no conclusions on the impact of screening experience can be drawn. Moreover, we only considered previous screening experience, not clinical expertise, as a potential effect modifier. Finally, we made simplified assumptions to enable the practical implementation of the study. For instance, various potential dependencies were not further considered. As stated, dependencies between the single screenings may exist, as the same screener was involved in several screenings.

### Practical applicability and future research

Further research is needed to determine the reliability of prioritization tools for study selection in daily practice. The variants of prioritization approaches mentioned above [25, 26] should be further examined, including the impact of the number of citations that have to be screened and/ or the number of studies included. Potential areas of use of prioritization tools include HTAs with many hits, many relevant studies, tight deadlines as well as HTA updates.

A double-screening approach seems justified in many cases. In the case of very complex projects where a large number of hits and relevant studies are expected, a single-screening approach may be appropriate for reasons of efficiency. However, over inclusive screeners could add workload to the full-text level. In addition, single screening should always be accompanied with searching additional sources. Therefore, ressource savings are questionable. If a search yields only a few hits, it is questionable whether a single-screening approach is appropriate. To reduce the screening burden, the combination of prioritization and either single- or double-screening, depending on the number of hits, might be an alternative. These combinations should be further evaluated.

## Conclusion

With regard to the question as to whether the use of the Rayyan or EPPI Reviewer tools for prioritizing the results of study selection increases efficiency, we found that the latter tool identified relevant citations earlier in the screening process. The potential reduction in resources through prioritization needs to be balanced against the greater uncertainty of results. Overall, our findings seem promising and we will continue to test how prioritization tools can be applied in future HTAs.

With regard to the question as to how accurately a single-screening approach identifies relevant studies, we found that single screening would have been sufficient to identify all studies relevant to the HTA conclusion. However, this requires the screening of further information sources. It also needs to be considered that the credibility of an HTA may be questioned if studies are missing, even if they are not relevant to the HTA conclusion. The resources required to search further information sources as well as over-inclusive screeners could add workload to the single-screening approach and may well outweigh the resources required for double-screening.

## Data Availability

The datasets analyzed during the current study are available from the corresponding author on reasonable request.

## Appendix 1

Characteristics of the 33 screening processes

**Table:**

No	Study type (1 = RCT 2 = non-RCT)	Experience (1 = < 3 screenings, 2 = 3–10 screenings, 3 = > 10 screenings)	Proportion of publications included correctly (after title/abstract screening)	Proportion of publications included correctly (after full-text screening)
1	1	1	100%	100%
2	1	2	100%	100%
3	1	2	100%	100%
4	1	2	100%	86%
5	1	3	71%	71%
6	1	2	71%	71%
7	2	1	100%	100%
8	2	2	0%	0%
9	2	3	100%	100%
10	1	3	96%	96%
11	1	3	91%	74%
12	1	3	96%	87%
13	1	2	100%	100%
14	1	2	96%	92%
15	1	3	71%	67%
16	1	2	100%	100%
17	1	2	96%	92%
18	1	2	65%	62%
19	2	3	100%	100%
20	2	3	100%	100%
21	2	2	100%	100%
22	2	3	100%	100%
23	2	3	100%	100%
24	2	2	100%	100%
25	1	3	100%	92%
26	1	3	92%	92%
27	1	1	92%	67%
28	1	2	96%	96%
29	1	3	92%	80%
30	1	3	92%	80%
31	1	3	100%	100%
32	1	2	100%	100%
33	1	3	100%	100%

## Appendix 2

Overview of individual results for EPPI Reviewer/Rayyan

**Table:**

**EPPI reviewer**				
**Screening**^**a**^	**STOPP 25%**	**STOPP 50%**	**STOPP 75%**	**Study type**
Screening No. 12	57%	91%	91%	RCT
Screening No. 15	42%	60%	71%	RCT
Screening No. 16	85%	100%	100%	RCT
Screening No. 7	100%	100%	100%	Non-RCT
Screening No. 19	100%	100%	100%	Non-RCT
Screening No. 26	83%	92%	92%	RCT
Screening No. 30	84%	92%	92%	RCT
Screening No. 32	90%	100%	100%	RCT
Screening No. 6	14%	43%	86%	RCT
Screening No. 24	100%	100%	100%	Non-RCT
**Rayyan**				
	**STOPP 25%**	**STOPP 50%**	**STOPP 75%**	**Study type**
Screening No. 5	29%	43%	43%	RCT
Screening No. 13	69%	92%	96%	RCT
Screening No. 21	50%	50%	100%	Non-RCT
Screening No. 8	0%	0%	0%	Non-RCT
Screening No. 10	74%	87%	96%	RCT
Screening No. 25	83%	100%	100%	RCT
Screening No. 29	68%	88%	92%	RCT

^**a**^The data were transmitted anonymously; therefore, the individual results cannot be assigned to any project
